# Influence of Curing-Induced Adhesive Behavior on Joint Formation and Mechanical Performance in CFRP/Al Hybrid Joints

**DOI:** 10.3390/polym18101252

**Published:** 2026-05-21

**Authors:** Chan Gon Park, Min Woo Park, Byeong Ju Jin, Ji Yeon Shim

**Affiliations:** 1 Carbon & Light Materials Application R&D Group, Korea Institute of Industrial Technology, Jeonju 54853, Republic of Korea; 2 R&D Center, WELMATE Co., Ltd., Cheonan-si 31217, Republic of Korea; 3 AI & Mechanical System Center, Advanced Technology Research Institute, 175-28, Goan-ro 51beon-gil, Cheoin-gu, Yongin-si 17047, Gyeonggi-do, Republic of Korea

**Keywords:** adhesive curing, CFRP/aluminum hybrid joints, adhesive behavior, joint formation, mechanical performance

## Abstract

This study investigates how the adhesive curing state before riveting influences material flow during riveting, joint formation, and the mechanical performance of CFRP/aluminum hybrid joints. Hybrid joints were fabricated in a single-lap configuration using electromagnetic self-piercing riveting (E-SPR) at curing times of 0, 20, 40, 60, and 80 min, and the adhesive distribution, joint geometry, load–displacement behavior, energy absorption, and failure mode were examined. As curing time increased, adhesive squeeze-out decreased and adhesive displacement during riveting was progressively restricted, leaving more adhesive near the contact point. Consequently, the head height increased from 0.12 to 0.21 mm, whereas the interlock distance decreased from 0.67 to 0.54 mm. In the bonded region, the peak load increased with curing time, and a peak load of 11.15 kN was observed at 40 min, indicating an increased contribution of the adhesive layer. In contrast, the load in the riveted region decreased at 60 and 80 min because the increased resistance of the adhesive interlayer limited the rivet deformation and mechanical interlocking. A maximum energy absorption of 32.13 J was observed at 40 min, where the joint exhibited relative contributions of the adhesive and the rivet. Failure analysis showed bearing failure at 40 min, whereas rivet pull-out was observed at 60 min, consistent with the curing-dependent changes in joint formation. These results indicate that curing-induced changes in adhesive behavior govern the interaction between adhesive flow and rivet deformation, thereby influencing joint formation and mechanical performance.

## 1. Introduction

Vehicle weight reduction is required to meet CO_2_ emission regulations and carbon-neutrality targets in the automotive and aerospace industries. To meet these requirements, multimaterial body-in-white (BIW) architectures that combine high-strength steels, aluminum alloys, and carbon fiber-reinforced polymers (CFRP) are used. For example, replacing a conventional steel BIW with an aluminum-intensive structure reduces vehicle mass by approximately 150–250 kg [1]. Within such multi-material systems, CFRP offers high specific stiffness and strength, enabling mass reduction without sacrificing load-bearing performance, while aluminum alloys provide a cost-effective balance of formability and structural efficiency. Therefore, their combination is well suited for load-bearing automotive structural panels and aerospace secondary structures [2,3]. However, the practical implementation of such multi-material assemblies depends on the joining technology. The elastic modulus, coefficient of thermal expansion (CTE), and surface chemistry of CFRP and aluminum differ substantially, and these differences introduce mechanical and physicochemical challenges that place strict requirements on the joining method [2].

Adhesive bonding has been widely adopted for CFRP–aluminum assemblies because it uniformly distributes the load across the bond area and avoids direct fiber damage. However, in practice, purely adhesive joints have several limitations. Under out-of-plane or eccentric loading, peel stresses concentrate at bondline terminations, and the CTE mismatch between CFRP and aluminum generates thermal residual stresses during adhesive curing that can initiate interfacial damage before service loading [3]. Hygrothermal aging further degrades adhesive shear strength through plasticization of the polymer matrix and deterioration of the adhesive–substrate interface, and these effects become more severe as the adhesive’s service temperature approaches the glass transition temperature (Tg) [4,5]. Conversely, mechanical fastening approaches, including solid riveting, bolting, and self-piercing riveting (SPR), avoid adhesive degradation but introduce different failure mechanisms. Because thermoset CFRP is quasi-brittle, the through-thickness rivet penetration in conventional SPR severs fibers, initiates delamination in the plies adjacent to the rivet hole, and generates subsurface matrix cracking, thereby reducing static bearing strength and fatigue resistance under cyclic loading. Furthermore, the electrochemical potential difference between electrically conductive carbon fibers and aluminum alloys establishes a galvanic couple at the fastener–composite interface, accelerating galvanic corrosion in the presence of moisture [5,6]. In hybrid bonded–riveted configurations, the presence of an adhesive layer partially mitigates this issue by acting as a physical barrier between dissimilar materials. Pan et al. [7] demonstrated that the modification of epoxy adhesives can significantly reduce galvanic current density and improve corrosion resistance in CFRP/aluminum hybrid joints, highlighting that the adhesive functions as a barrier against galvanic corrosion, beyond load transfer. Similar approaches have also demonstrated that modifications in epoxy systems can significantly influence adhesion behavior and joint performance [8]. These limitations indicate that neither adhesive-only nor mechanical-fastening-only configurations can satisfy the static strength, fatigue resistance, and environmental durability requirements of structural CFRP–aluminum joints. In addition, the curing process alters the adhesive’s viscoelastic behavior and flow resistance, which can significantly influence its displacement and distribution during the riveting process.

Previous studies have shown that defects in the adhesive bondline, including an improperly cured bondline, can significantly reduce the static strength and fatigue life of hybrid joints [9]. In addition, the curing state at the time of joining has been identified as a critical parameter, since unfavorable rivet insertion timing during curing can lead to inferior joint resistance in hybrid joints [10].

As the adhesive cures, its rheological condition and chemical reactivity evolve continuously, which can influence the quality of the joint and its resulting mechanical properties during joining [10]. Consequently, changes in the curing state can alter the flow behavior of the adhesive during joining. In addition, in dissimilar-adherend joints, adhesive-related parameters such as bondline thickness are known to affect fracture behavior and failure load [5]. Therefore, the curing state of the adhesive can be an important factor influencing the final performance of hybrid joints. Hybrid joining, in which structural adhesives and mechanical fasteners are applied simultaneously, addresses these limitations through a complementary load-sharing mechanism. Recent studies have explored hybrid joining strategies combining mechanical interlocking and adhesive bonding to improve joint performance [11]. In particular, Sam-Daliri et al. [12] compared adhesive bonding and co-curing techniques in composite joints, demonstrating that bonding strategy and processing conditions significantly influence joint strength and failure behavior. Under applied loading, the adhesive layer transfers in-plane shear load across the full bond area, while the fasteners suppress peel stress at the bondline terminations and provide a fail-safe load path that arrests crack propagation after adhesive fracture.

Previous studies on hybrid CFRP–aluminum joints can be grouped into fatigue-oriented investigations, defect-tolerance studies, and parametric optimization analyses. Fatigue-oriented studies demonstrated load sharing between adhesive layers and mechanical fasteners. He et al. [13] used digital image correlation (DIC) to quantify this load redistribution during fatigue cycling, demonstrating that the mechanical fastener’s contribution to joint stiffness increases progressively as the adhesive accumulates damage, thereby extending fatigue life beyond both riveted-only and bonded-only configurations. Defect-tolerance studies have highlighted the robustness of hybrid joints under imperfect bonding conditions. Chowdhury et al. [9] confirmed the effectiveness of hybrid CFRP joints under both static and fatigue loading in an aircraft-structure context, including intentional adhesive defects, where the rivets’ fail-safe role was particularly significant. Gamdani et al. [14] further demonstrated that hybrid bonded–bolted joints exhibit a two-stage fatigue response, in which progressive adhesive debonding is followed by load transfer to the mechanical fasteners, resulting in significantly longer fatigue life than purely bolted joints. Furthermore, parametric investigations have focused on the influence of joint design variables such as rivet type, adhesive layer thickness, and substrate material combination on joint strength and energy absorption [15,16]. Temperature-dependent behavior has also been investigated because automotive structures experience a wide range of service temperatures. In addition, Dong et al. [17] investigated the bond behavior of CFRP-to-steel interfaces using single-lap joints under varying temperature conditions, demonstrating that changes in adhesive properties significantly influence interfacial stress transfer and failure behavior. Chen et al. [18] tested hybrid bonded–riveted CFRP–aluminum joints from −30 to 100 °C and showed that joint strength decreased by 26% at −30 °C and by 43% at 100 °C relative to room temperature, while an increase of approximately 8% was observed at 60 °C. The DSC analysis identified the glass transition temperatures of the polyurethane adhesive (75 °C) and the CFRP resin matrix (121 °C) as the material parameters governing these transitions. Failure sequences across these studies consistently followed a multi-stage progression: cohesive fracture of the adhesive layer, bearing damage and delamination of the CFRP laminate, and shear fracture of the rivet.

Despite these investigations, a primary limitation of previous studies on hybrid joints is the exclusive use of conventional hydraulic SPR as the mechanical fastening process. In these processes, the punch force required to drive the semi-hollow rivet through the CFRP upper layer imposes a compressive load perpendicular to the fiber layers. This loading results in delamination, matrix cracking, and fiber cutting near the rivet hole, damage that cannot be fully eliminated by rivet geometry or die-shape optimization alone [5,6]. Vorderbrüggen et al. [19] further elucidated this damage mechanism, showing that self-piercing riveting induces not only initial fiber breakage and inter-fiber failure but also progressive delamination under subsequent thermal loading conditions representative of automotive paint processes. Thermally induced stresses arising from the mismatch in thermal expansion coefficients can significantly amplify pre-existing damage around the rivet region.

Therefore, several modified self-piercing riveting processes have been proposed to improve joint quality and reduce damage in composite materials. Wang et al. [20] developed a post-curing self-piercing riveting (PC-SPR) process in which CFRP–aluminum assemblies were cured after riveting, resulting in improved joint strength and reduced delamination around the rivet region. Zhang et al. [21] proposed a hemming self-piercing riveting (H-SPR) technique that forms a mechanical hemming structure through controlled rivet deformation, significantly enhancing load capacity and energy absorption compared with conventional SPR joints. More recently, Xu et al. [22] introduced an electromagnetic pre-holed self-piercing riveting (EPH-SPR) approach that utilizes electromagnetic loading to achieve more uniform rivet deformation and reduce CFRP damage. E-SPR has been reported to involve high-velocity rivet insertion, which can influence deformation localization during rivet piercing and thereby affect joint formation and resulting joint characteristics [23,24].

Jin et al. [25] reported that the use of a flat die and a novel semi-solid rivet reduced CFRP damage compared with a traditional semi-hollow rivet joint, while increasing peak load and energy absorption by 18.3% and 13.5%, respectively. Fiore et al. [10] investigated the effect of curing time on the performance of Al/GFRP hybrid joints produced by combining co-curing and SPR. Previous studies have shown that the curing state of the adhesive at the time of riveting affects the mechanical performance of hybrid bonded–riveted joints. However, how curing-time-dependent changes in adhesive behavior during riveting govern adhesive squeeze-out and residual distribution, joint formation characteristics such as rivet deformation and interlock, and the mechanical performance of CFRP/aluminum hybrid joints has not been clearly established. Therefore, this study investigates how the adhesive curing state before riveting influences material flow during riveting, joint formation, and the mechanical performance of CFRP/aluminum hybrid joints.

## 2. Materials and Methods

### 2.1. Materials

CFRP (KompoGTe^®^, Kolon Plastics Inc., Gimcheon, Republic of Korea) and aluminum alloy (Al 6061-T6) were used as adherends, and a structural epoxy adhesive (TEROSON EP 5075 SB, Henkel, Düsseldorf, Germany) was utilized for hybrid joining. The adhesive is a two-component epoxy system with a mixing ratio of 2:1 (resin:hardener, by volume). The CFRP consisted of 10 prepregs of T700 carbon fiber reinforced polyamide 6 (thermoplastic matrix) with a symmetric layup configuration of [0/90/0/90/0]_2s_. Both the CFRP and aluminum sheets had a nominal thickness of 2.0 mm. The mechanical properties of the CFRP were provided by the manufacturer (EZ Composite, Gimje, Republic of Korea), those of A6061-T6 were obtained from the ASM Handbook [26], and those of the rivet were obtained from literature [27]. Table 1 summarizes the mechanical properties of the CFRP, aluminum sheet, and rivet used in this study.

### 2.2. Preparation of Specimens and Adhesive Curing

**Figure 1:**
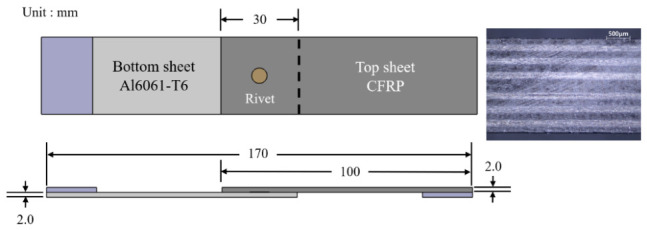
Single-lap shear specimen geometry and dimensions.

The sheets were cut into rectangular specimens of 100 × 30 mm using a waterjet cutting system (T500-3015, TOPS Co., Ltd., Gimhae, Republic of Korea). The bonding surfaces of both CFRP and aluminum sheets were manually abraded with 320-grit abrasive paper, ultrasonically cleaned for 3 min, and dried in a vacuum oven at 80 °C for 3 h. Figure 1 illustrates the geometry and dimensions of the single-lap shear specimen. The single-lap joint configuration was designed based on ASTM D1002 [28]. To minimize load misalignment, tabs were attached to both specimen ends. A structural adhesive was applied to prepare adhesive-only single-lap joints with a controlled thickness of 0.2 mm to investigate the joining characteristics under different adhesive curing conditions. The adhesive layer thickness was controlled using a structural adhesive containing pre-dispersed glass beads with a nominal diameter of approximately 0.20 mm, which act as spacers to ensure a uniform bond-line thickness. As a result, the bond-line thickness was physically constrained and maintained at approximately 0.2 mm, regardless of the applied pressure during joining. These specimens were cured at 60 °C for different curing times (0, 20, 40, 60, and 80 min). A universal testing machine (Instron, Norwood, MA, USA) equipped with a 100 kN load cell at a constant crosshead speed of 13 mm/min was employed for the lap shear tests. Accordingly, five specific curing conditions were selected for the subsequent hybrid joining process. Five specimens were tested for each experimental condition to ensure consistency.

### 2.3. Hybrid Joining Process

**Figure 2:**
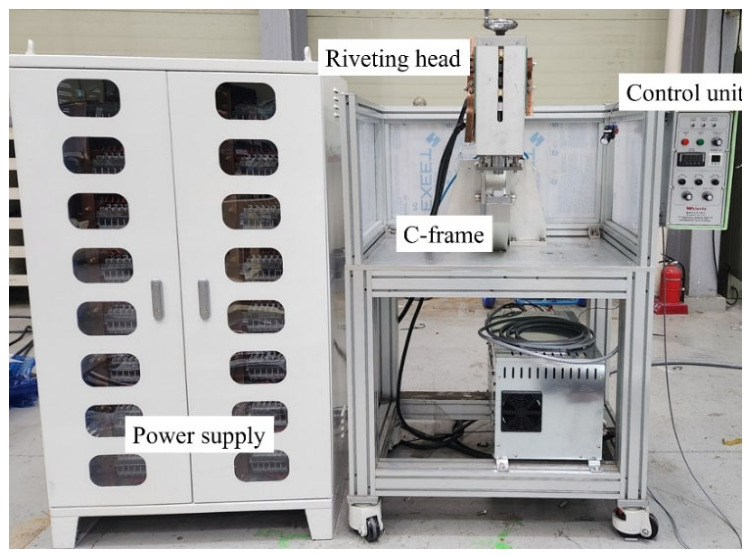
Configuration of the E-SPR system.

The E-SPR process was conducted utilizing an electromagnetic riveting system (WELMATE Co., Ltd., Cheonan, Republic of Korea). The system consists of a riveting head, C-frame, power supply, and control unit. The riveting head includes an electromagnetic coil for pulse generation and a punch for rivet insertion, and the C-frame was fabricated from aluminum alloy to reduce weight. Figure 2 illustrates the overall configuration of the electromagnetic riveting system used in this study. The power supply is equipped with 192 capacitors (225 μF each), providing a maximum charging voltage and peak energy of 272 V and 10 kJ, respectively. The riveting process was conducted at a discharge energy of 4.0 kJ, which was determined via preliminary experiments to ensure full penetration and proper interlocking of the CFRP/Al combination. The initial clamping force was set to 3.5 kN.

**Figure 3:**
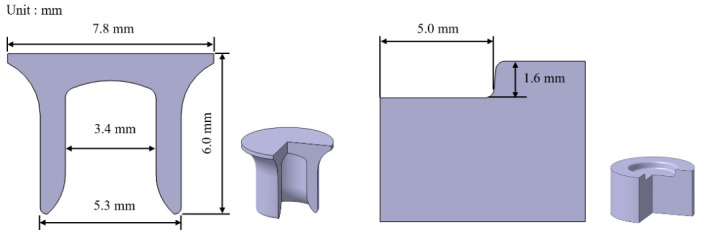
Geometry of the rivet and die.

Boron steel rivets (C-type, Böllhoff, Bielefeld, Germany) with a length of 6 mm and an Almac coating (480 ± 30 HV) were used with a flat-bottom die (FM-type, Böllhoff, Bielefeld, Germany). Figure 3 illustrates the rivet and die geometry used in the E-SPR process. All process parameters were kept constant for all experimental conditions. The mechanical properties of the hybrid joints were evaluated through lap shear tests under the same conditions described above. Five specimens were tested for each curing condition.

### 2.4. Characterization

The calorimetric properties of the structural adhesive were studied using differential scanning calorimetry (DSC; DSC204F1, NETZSCH, Selb, Germany). Approximately 30 mg of adhesive samples were utilized in sealed aluminum pans (diameter: 5.9 mm, height: 1.75 mm, capacity: 40 µL). The aim of this analysis was to evaluate the residual heat reaction of the adhesive as a function of the pre-curing time, specifically, the adhesive’s residual capacity to cure or chemical reactivity. For this reason, seven experiments were performed under a nitrogen atmosphere in temperature ramp mode from room temperature up to 200 °C, with a temperature rate of 10 °C/min. In particular, the ramps were carried out by initially leaving the structural adhesive to cure at 60 °C for 0 min (i.e., the initial time of curing process), 20, 40, 60, 80, 100, and 120 min. These tests were used to define the total heat reaction associated with the full conversion of the adhesive under the aforementioned curing process conditions. To further examine the effect of adhesive mass on the DSC response, additional measurements were performed with adhesive samples of 20 and 10 mg at curing times of 0, 40, and 80 min, under identical conditions. The rheological behavior of the structural adhesive during curing was characterized using a rotational rheometer (MCR 102e, Anton Paar, Graz, Austria) equipped with a disposable parallel-plate geometry (25 mm diameter). The gap between the plates was set to 0.5 mm. Time-sweep measurements were conducted at 60 °C under isothermal conditions in strain-controlled oscillation mode (TruStrain®) at 1 Hz and 0.5% strain for 7000 s. The storage modulus (G′) and loss modulus (G″) were recorded as a function of time.

The hybrid joints were sectioned along the rivet centerline parallel to the loading direction using a diamond wafer blade for microstructural analysis. The cross-sections were examined using an optical microscope (GX51-N213B, Olympus, Tokyo, Japan) to measure geometric parameters, including rivet head height, interlock distance, and bottom thickness. The adhesive distribution and interfacial thickness were evaluated based on optical images of cross-sections. The fracture surfaces of the hybrid joints were further examined using scanning electron microscopy (SEM; JSM-7100F, JEOL, Tokyo, Japan) to analyze the fracture morphology. All data are presented as mean ± standard deviation (*n* = 5). Statistical significance was evaluated by one-way analysis of variance followed by Tukey’s post hoc test (*p* < 0.05).

## 3. Results and Discussion

### 3.1. Validation of the Curing Condition

**Figure 4:**
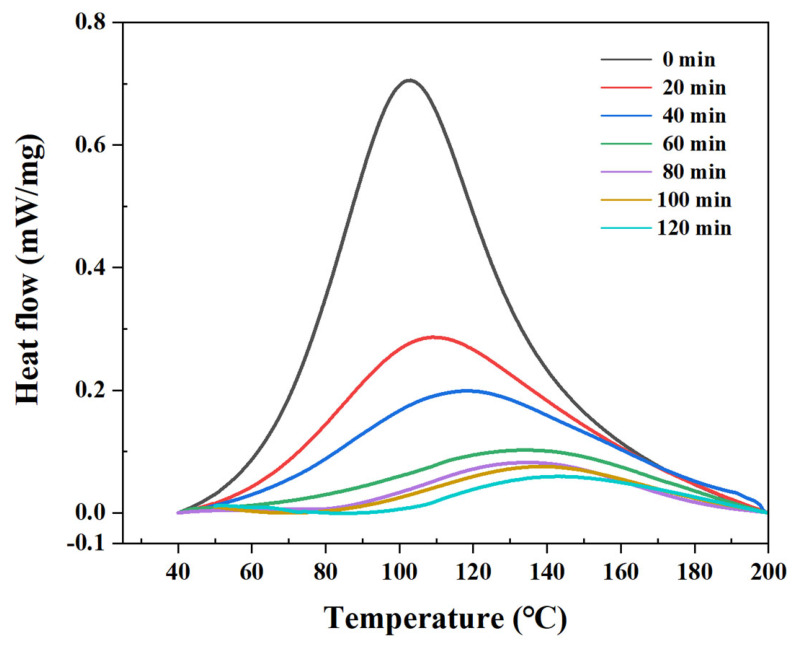
Heat flow–temperature curves of the adhesive at different curing times.

Seven experiments were performed to evaluate the residual reaction heat or chemical reactivity of the adhesive at different curing times. Figure 4 presents the heat flow versus temperature curves of the adhesive under all the investigated conditions. Table 2 summarizes the total reaction heat values and exothermic peak temperatures. The total reaction heat decreased from 242.89 to 21.56 J/g at 0 and 120 min, respectively, indicating a reduction in the adhesive’s residual chemical reactivity as curing proceeds. The maximum temperature of the exothermic peak increased with the curing time, shifting from 102.61 to 143.50 °C at 0 and 120 min, respectively. The total reaction heat decreased significantly up to 60 min, indicating that the adhesive reached a substantially cured state. From 80 to 120 min, only a small decrease in the total reaction heat was observed, indicating that the curing reaction was nearly complete. This decrease in the residual reaction heat indicates an increase in the degree of cure, which is associated with the increased resistance to deformation and reduced adhesive flowability.

**Figure 5:**
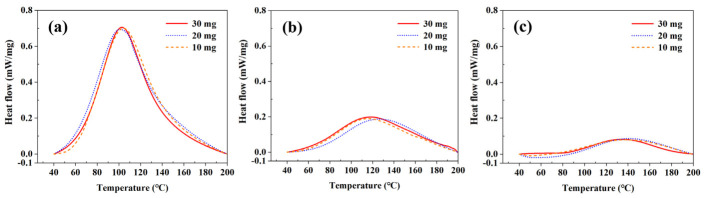
Heat flow–temperature curves of the adhesive at different adhesive masses and curing times: (**a**) 0 min, (**b**) 40 min, and (**c**) 80 min.

Since the adhesive thickness can locally vary during the riveting process owing to the curing-induced changes in adhesive flowability, additional DSC measurements were performed using different adhesive masses of 30, 20, and 10 mg to simulate variations in adhesive thickness, at curing times of 0, 40, and 80 min. Figure 5 presents the heat flow versus temperature curves of the adhesive obtained at the different adhesive masses, and Table 3 summarizes the corresponding total reaction heat values and exothermic peak temperatures. At each curing time, the heat flow curves obtained at the three adhesive masses exhibited nearly identical profiles, and the overall decrease in total reaction heat with increasing curing time was consistently preserved. This confirms that the DSC-based interpretation of the curing state is not substantially affected by adhesive thickness variation.

**Figure 6:**
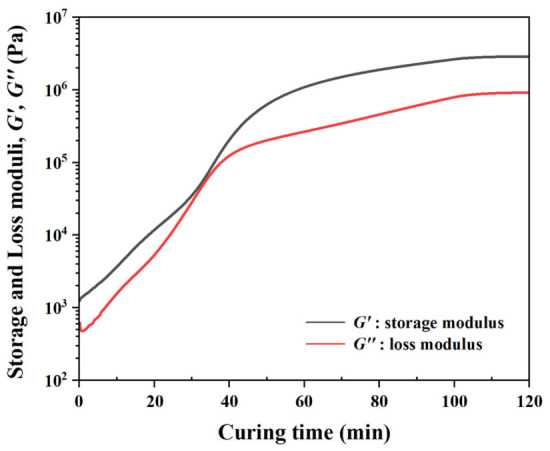
G′ and G″ versus time for the structural adhesive measured at 60 °C.

The isothermal curing behavior of the structural adhesive was characterized at 60 °C by monitoring G′ and G″ as a function of time, as shown in Figure 6. Throughout the measurement, G′ remained higher than G″, and no G′−G″ crossover was observed. Both G′ and G″ increased continuously with time. In particular, G′ increased markedly after approximately 2100 s (≈35 min) and up to 3500 s (≈60 min), from the order of 10^5^ Pa to above 10^6^ Pa, indicating rapid curing-induced stiffening of the adhesive. Around 60 min, the rate of increase in G′ decreased, suggesting that the adhesive approached a highly stiffened state. These results indicate that curing at 60 °C resulted in a significant increase in the stiffness of the adhesive.

**Figure 7:**
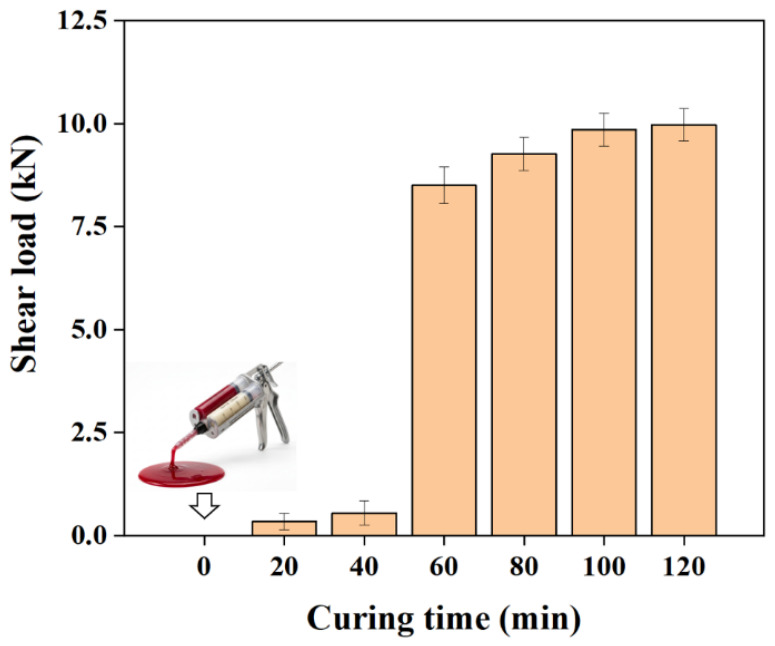
Adhesive-only lap shear load at different curing times.

The mechanical state of the adhesive at each stage was characterized through single-lap shear tests using adhesive-only specimens. Figure 7 presents the results of the adhesive-only lap shear load. The adhesive-only lap shear load clearly depended on the curing time. No measurable load was obtained at 0 min, whereas only a negligible load was observed at 20 min. The load increased sharply to 8.50 kN at 60 min, which was significantly higher than those at 0, 20, and 40 min (*p* < 0.05). A further increase was observed at 80 min, when the load was 9.26 kN. No substantial increase was observed beyond 80 min, with comparable values in 100 and 120 min, confirming a plateau.

**Figure 8:**
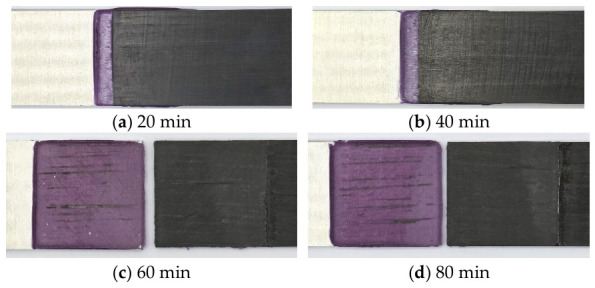
Adhesive-only joints after lap shear tests at different curing times. (the purple area represents the adhesive).

Figure 8 illustrates the adhesive-only joints after the lap shear tests at different curing times. No distinct fracture features were observed at 20 and 40 min, and the top sheet appeared to slide over the adhesive layer. A slightly more continuous adhesive layer was observed at 40 min than at 20 min. At 60 min, the adhesive was clearly observed on the bottom sheet, indicating mixed failure. A similar surface condition was observed at 80 min, with more carbon fibers remaining on the adhesive surface than at 60 min, indicating mixed failure with fiber-tear features.

Based on these results, five curing times of 0, 20, 40, 60, and 80 min were selected, while the 100 and 120 min conditions were excluded due to similar curing behavior and comparable lap shear responses. Table 4 summarizes the corresponding curing stages. These results indicate that the curing state before riveting affects the deformation behavior and load-carrying capability of the adhesive. This difference is expected to influence adhesive flow during riveting and the subsequent mechanical response of the hybrid joint.

### 3.2. Load–Displacement Behavior of Hybrid Joint

**Figure 9:**
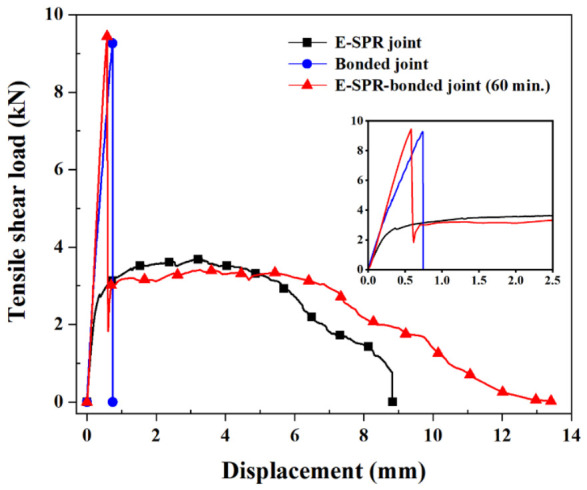
Shear load–displacement curves of riveted joints, bonded joints, and hybrid joints in 60 min.

Figure 9 illustrates the load–displacement behavior of the adhesive-only joint cured for 80 min, the E-SPR-only joint, and the hybrid joint fabricated at 60 min. For the adhesive-only joint, the load increased to approximately 9.26 kN and then dropped sharply. The adhesive layer carried the load and was not maintained after failure, indicating brittle behavior. The load of the E-SPR-only joint increased to approximately 3.69 kN and was sustained over a larger displacement range. The hybrid joint exhibited a distinct two-stage load–displacement response. The first peak load of approximately 9.45 kN was comparable to that of the adhesive-only joint, indicating that the adhesive layer mainly transferred the initial load. After the first peak, the load dropped sharply due to the adhesive layer failure. Owing to the single-lap geometry, secondary bending generates peel forces at the overlap ends. The adhesive layer primarily carries the distributed shear load and partially suppresses peel deformation. In contrast, the rivet provides localized load transfer and structural constraint through bearing contact, interlock distance, clamping force, and friction, contributing to additional shear resistance and peeling resistance. The load was transferred to the rivet, resulting in a second peak of approximately 3.41 kN and a subsequent plateau region similar to that of the E-SPR-only joint after adhesive failure [29]. However, the second peak load of the hybrid joint was slightly lower than that of the E-SPR-only joint, which is attributed to the prior contribution of the adhesive layer during deformation. These results indicate that the curing-induced behavior of the adhesive governs load transfer in the hybrid joint, which influences deformation during riveting and determines the resulting interlock geometry.

### 3.3. Effect of Curing Conditions on Hybrid Joints

(1) Joint formation

**Figure 10:**
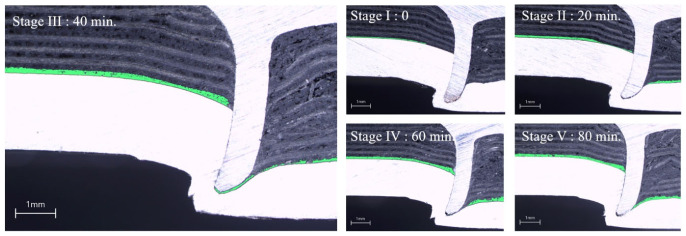
Adhesive layer distribution in hybrid joint cross-sections at different curing times.

The adhesive applied before riveting is displaced by the compressive pressure generated during rivet insertion in hybrid joining. Therefore, the curing time before riveting affects the final adhesive distribution along the joint interface. Figure 10 illustrates the adhesive distribution along the joint interface at different curing times, with the adhesive layer highlighted in green. As the curing time increased, the adhesive distribution changed, indicating that the adhesive responded differently to the riveting pressure during the E-SPR process. Most of the adhesive was squeezed out of the joint interface at 0 min (without curing), and no continuous adhesive layer was observed near the contact point. After 20 min of curing, a thin adhesive layer remained locally along the interface. Although most of the adhesive was still displaced during riveting, a small amount was retained, indicating the onset of resistance to squeeze-out. A different distribution was observed after 40 min. The adhesive layer became nonuniform, and accumulation was observed at a certain distance from the contact point. The adhesive’s outward displacement was partially restricted, resulting in localized thickening along the interface. At 60 and 80 min, the adhesive remained near the contact point with limited outward displacement, and a more continuous and thicker adhesive layer was observed near the rivet.

**Figure 11:**
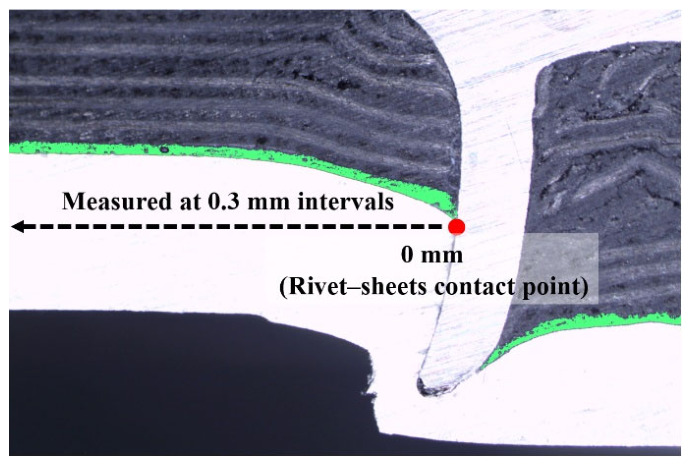
Measurement of adhesive layer thickness and its distribution along the joint interface at varying curing times.

Overall, as the curing time increased, the adhesive distribution changed from squeeze-out to localized accumulation and retention near the contact point. The observed variation in final adhesive thickness reflects curing-dependent changes in adhesive flow behavior during riveting. Figure 11 shows the adhesive thickness profiles quantified from the cross-sectional images. For visualization, the curves were smoothed using a moving average method, and the numerical results were consistent with the curing-time-dependent distribution observed. Near the center, the thickness approached zero at 0 min, indicating that the riveting pressure almost completely displaced the uncured adhesive. However, at 80 min, the thickness increased toward the center and reached approximately 0.33 mm, indicating that the adhesive was retained at the contact point. At 40 min, the adhesive was displaced at the contact point, whereas a thickness peak was observed away from the center, indicating that the adhesive was displaced from the contact point but was not completely squeezed out as curing progressed.

These results indicate that the resistance of the adhesive to squeeze-out during riveting governs the final interfacial thickness, which increases with curing time. This behavior is attributed to curing-induced stiffening of the adhesive, as confirmed by the rheological results shown in Figure 6, which demonstrate a significant increase in G′ during curing, leading to increased resistance to deformation and reduced adhesive flow during riveting.

**Figure 12:**
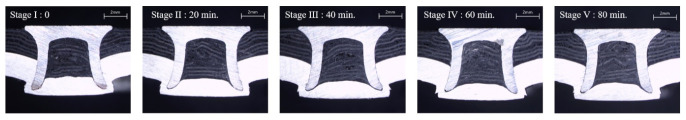
Cross-sections of hybrid joints at different curing times.

Figure 12 illustrates the cross-sections of the hybrid joints at different curing times. Variations in head height, interlock distance, and bottom thickness are observed with curing time under the same fastening energy.

**Figure 13:**
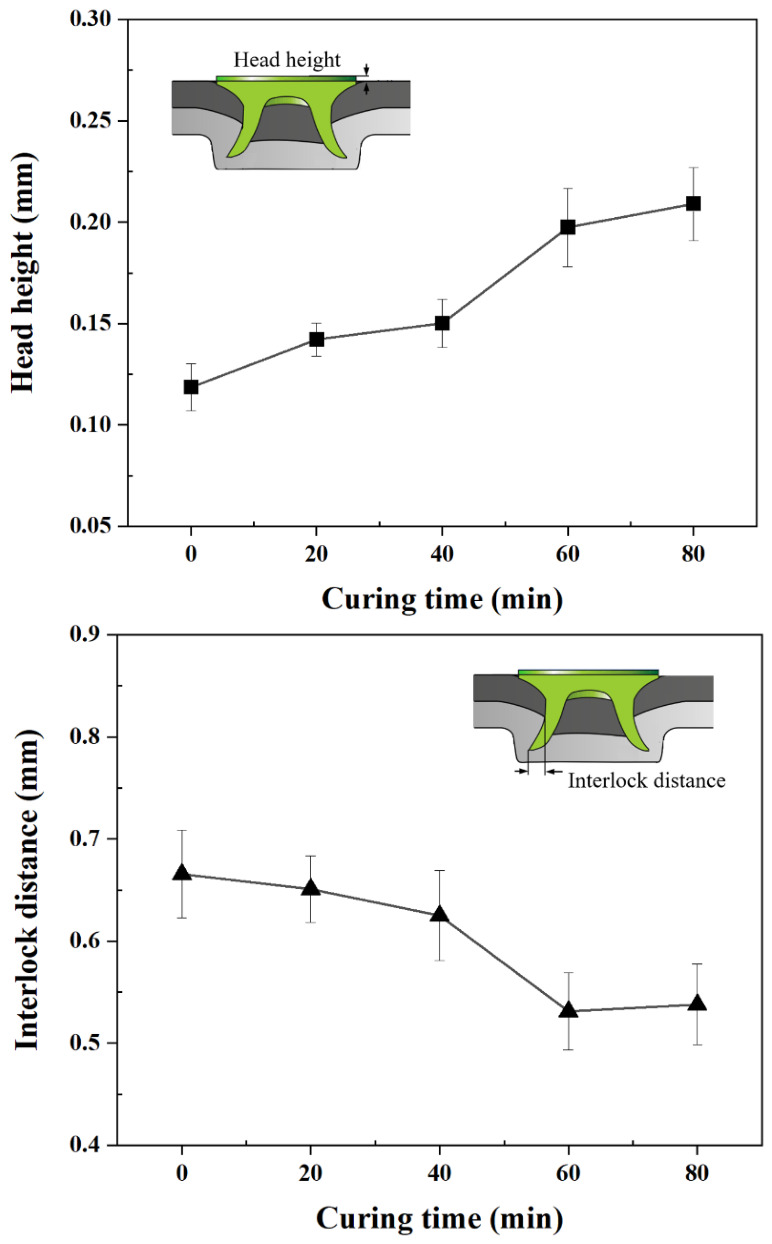

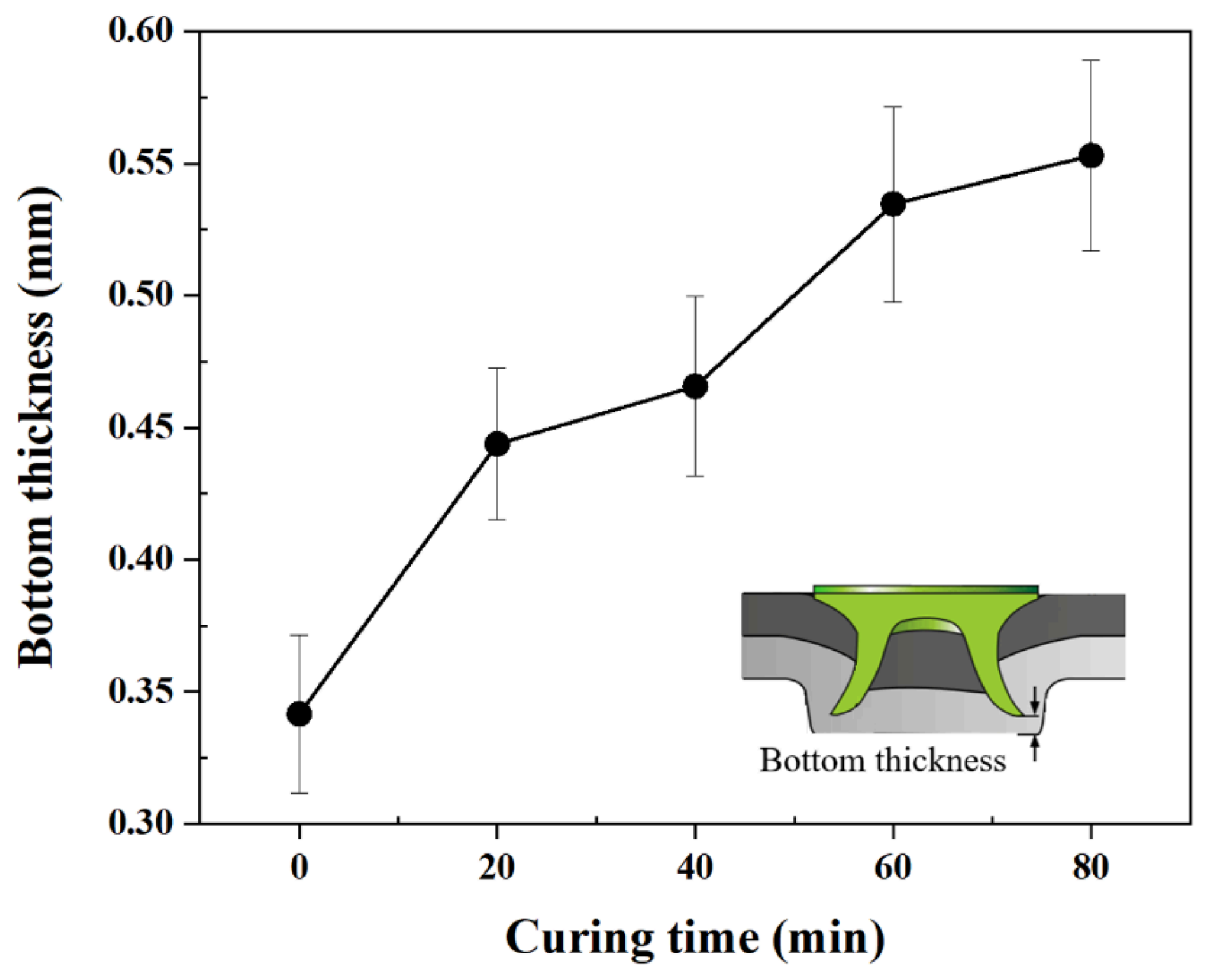
Variations in head height, interlock distance, and bottom thickness with curing time.

Figure 13 shows the variations in the head height, interlock distance, and bottom thickness with the curing time. The head height increased from 0.12 mm at 0 min to 0.15 mm at 40 min, followed by a larger increase to 0.20 mm at 60 min. Only a slight increase was observed from 60 to 80 min, from 0.20 to 0.21 mm. The liquid adhesive provides minimal resistance at 0 and 20 min. As curing progressed, the interlayer became more resistant to deformation, and the mechanical response approached a plateau at 60 and 80 min. Under the same riveting energy, this increased resistance limits rivet penetration, resulting in a higher head height.

Conversely, the interlock distance decreased from 0.66 mm at 0 min to 0.63 mm at 40 min and then to 0.53 mm at 60 min, which was significantly lower than that at 0 and 20 min (*p* < 0.05). The values remained nearly unchanged at 0.53 and 0.54 mm between 60 and 80 min. At 40 min, the adhesive begins to restrict the bottom sheet deformation. At 60 and 80 min, the increased mechanical response of the adhesive suppresses the radial expansion of the rivet legs, resulting in a smaller interlock than in the initial uncured state. The bottom thickness was 0.34 mm at 0 min, 0.44 mm at 20 min, 0.46 mm at 40 min, 0.53 mm at 60 min, and 0.55 mm at 80 min, consistent with the increase in head height and the decrease in interlock distance. This increase in bottom thickness is a geometric consequence of the reduced penetration and limited sheet deformation. These results indicate that the increased resistance of the adhesive limits deformation during riveting as curing progresses, resulting in increased head height and reduced interlock distance and altering the final joint formation.

(2) Mechanical behavior

**Figure 14:**
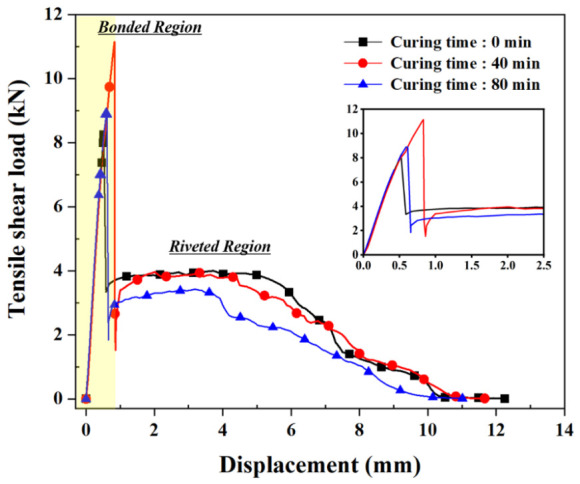
Shear load–displacement curves of hybrid joints at different curing times.

**Figure 15:**
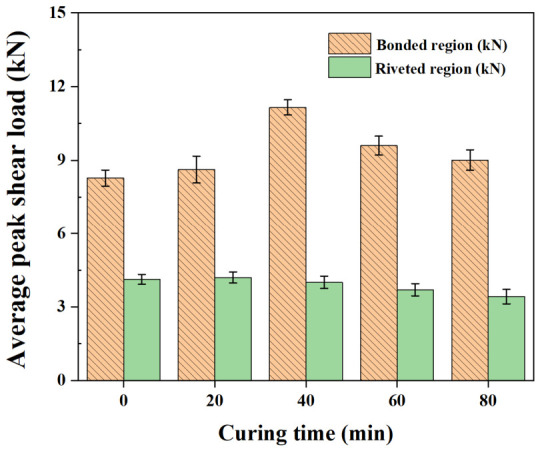
Average peak shear load of hybrid joints at various curing times.

Figure 14 shows the load–displacement curves of the hybrid joints at varying curing times. The overall behavior can be divided into the initial bonded region and the riveted region at larger displacements. Figure 15 summarizes the average peak loads for the bonded and riveted regions. Unlike the adhesive-only response, the hybrid joint response is governed by the combined contributions of adhesive bonding and rivet interlocking. In the bonded region, the peak load increased with the curing time before riveting. A maximum peak load of 11.15 kN was observed at 40 min, which was significantly higher than those at other curing conditions (*p* < 0.05), and then decreased as curing progressed further. At 0 and 20 min, only a limited load was observed because the low-viscosity adhesive was almost entirely squeezed out of the interface during rivet insertion. The similar peak loads observed at 60 and 80 min (9.59 and 9.01 kN, respectively) are consistent with the plateau in adhesive mechanical properties observed in Section 3.1.

In contrast, a decrease in load in the riveted region was observed with increasing curing time, from 4.13 kN at 0 min to 3.42 kN at 80 min, with significantly lower values at 60 and 80 min than at 0 and 20 min (*p* < 0.05). This behavior is consistent with changes in joint formation, as increased head height and reduced interlock distance weaken the mechanical interlocking between the rivet and the bottom sheet. Consequently, both the frictional resistance between the rivet legs and the bottom sheet and the plastic work required for rivet pull-out are reduced. These results indicate that although the adhesive contribution is maximized at the intermediate curing state, the rivet contribution progressively decreases because of the reduced rivet interlocking due to the increased resistance of the adhesive interlayer during riveting. Accordingly, optimum joint performance was obtained at an intermediate curing condition, where the adhesive contribution was improved without a substantial loss of mechanical interlocking [12].

**Figure 16:**
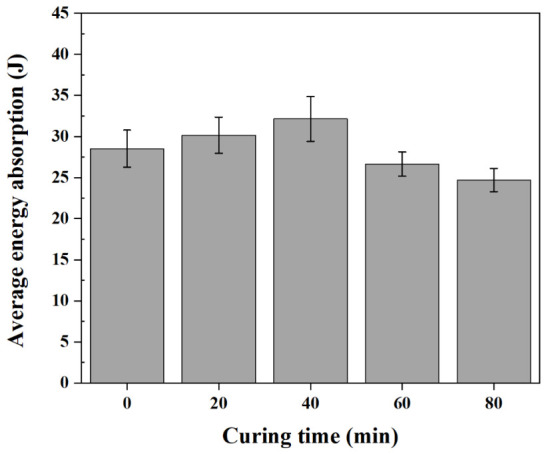
Average total energy absorption of hybrid joints at various curing times.

Figure 16 shows the total energy absorption of hybrid joints at various curing times. The total energy absorption of the hybrid joints increased from 28.51 J at 0 min to 32.13 J at 40 min and then decreased to 26.66 J at 60 min and 24.71 J at 80 min. A maximum energy absorption of 32.13 J was observed at 40 min, which was significantly higher than those at 60 and 80 min (*p* < 0.05). This behavior is mainly governed by riveted region deformation. Although the bonded region contributes to the peak load, the riveted region governs the overall behavior over a wider displacement range and has a greater influence on energy absorption. At advanced curing stages, the reduction in interlock distance limits the effective displacement during rivet pull-out, resulting in lower energy absorption. At 40 min, the adhesive contribution increased without a significant decrease in the rivet contribution.

Overall, the mechanical behavior of the hybrid joints is governed by the contributions of the adhesive and rivet, which are affected by the adhesive’s curing-dependent flow resistance during riveting. As curing progresses, the adhesive contribution increases, whereas the rivet contribution decreases, resulting in increased head height and reduced interlock distance and shifting the load–displacement curve.

**Figure 17:**
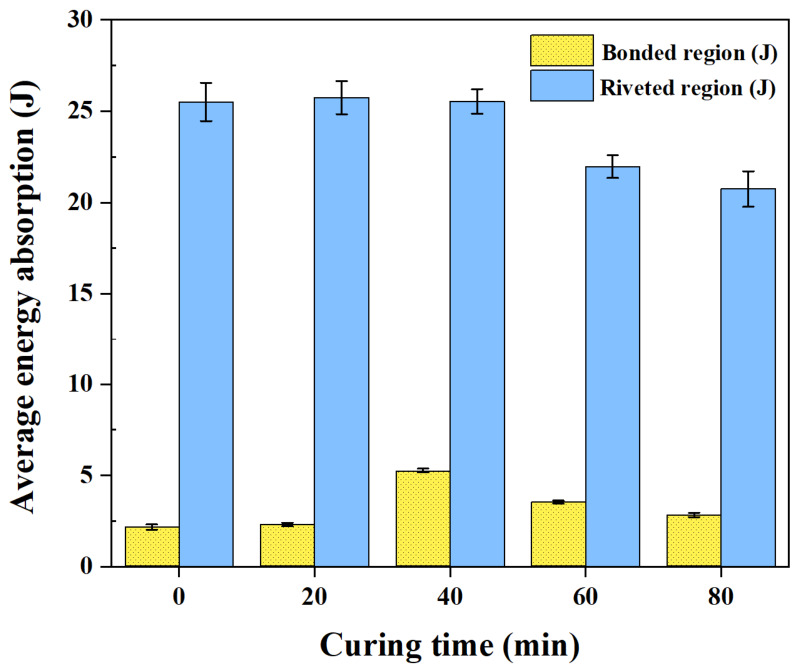
Average energy absorption in bonded and riveted regions as a function of curing time.

Figure 17 shows the energy absorption in the bonded and riveted regions as a function of curing time. In the bonded region, the energy absorption increased with curing time and reached a maximum at 40 min, followed by a decrease at 60 and 80 min. The maximum value at 40 min was significantly higher than those at the other curing conditions (*p* < 0.05). In contrast, the energy absorption in the riveted region remained nearly constant from 0 to 40 min and then decreased at 60 and 80 min. Significantly lower values were observed at 60 and 80 min compared to the earlier curing conditions (*p* < 0.05).

(3) Failure mode

**Figure 18:**
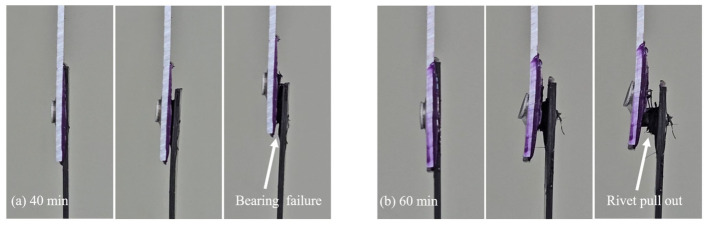
Failure modes of the hybrid joints at varying curing times: (**a**) overall and close-up views of bearing failure at 40 min, and (**b**) overall and close-up views of rivet pull-out at 60 min.

**Figure 19:**
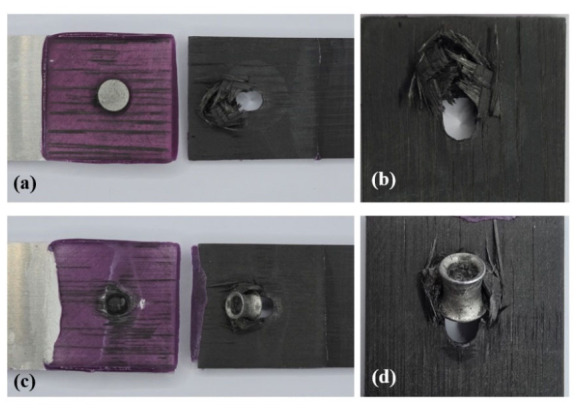
Final failure modes of the hybrid joints at varying curing times: (**a**,**b**) bearing failure at 40 min in overall and close-up views; (**c**,**d**) rivet pull-out at 60 min in overall and close-up views.

Figure 18 shows the failure process of the hybrid joints during tensile-shear loading at 40 and 60 min. At 40 min, a larger deformation was observed, and the bearing failure failed the joint. At 60 min, less deformation was observed, and the joint failed due to the pull-out of the rivet. Figure 19a–d presents the final failure modes at 40 and 60 min. This difference was related to the interlock formed during riveting and was consistent with the load reduction sustained in the riveted region and the decrease in total energy absorption at 60 min.

The hybrid joint primarily exhibited bearing failure in the bottom sheet at 40 min. Under this condition, the mechanical interlocking formed during riveting remained sufficient to maintain the engagement between the rivet and the bottom sheet after adhesive failure. Consequently, over a relatively large displacement range, the rivet continued to transfer load to the bottom sheet. The elongated hole and pronounced deformation around the joint indicate that substantial bearing deformation developed in the bottom sheet, resulting in large deformation and energy absorption. This failure morphology is consistent with the high-energy absorption observed at 40 min.

Conversely, the final failure mode changed to rivet pull-out with limited deformation of the bottom sheet at 60 min. This behavior is consistent with the joint formation described in Section 3.3. As curing progressed, the increased flow resistance of the adhesive layer during riveting limited the radial expansion of the rivet legs, resulting in increased head height and reduced interlock distance. The rivet could not sustain bearing deformation of the bottom sheet to the same extent because of this reduced interlock. Instead, the rivet was pulled out through the sheet, resulting in local enlargement and hole damage. Consequently, the effective deformation in the riveted region was reduced, resulting in reduced deformation and energy absorption. These observations indicate that the hybrid joint maintained sufficient interlock to sustain deformation in the riveted region after adhesive failure at 40 min. However, when the curing time was increased from 60 to 80 min, the increased resistance of the adhesive layer during riveting reduced the rivet interlocking and shifted the final failure mode toward pull-out, thereby decreasing the energy absorption of the joint.

**Figure 20:**
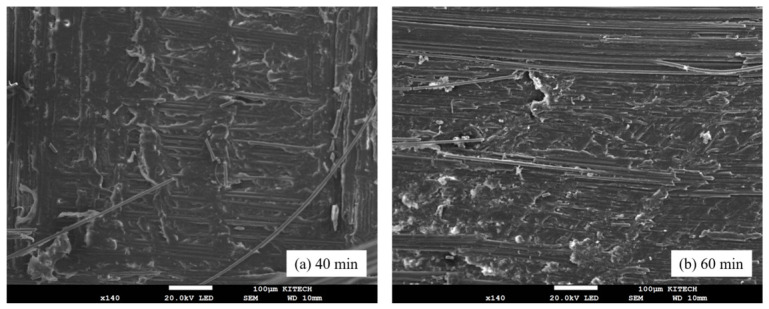
SEM images of fracture surfaces in the adhesive region at different curing conditions: (**a**) 40 min and (**b**) 60 min.

The fracture surfaces of the hybrid joints were analyzed. Distinct differences in fracture morphology were observed between the 40 and 60 min conditions, as shown in Figure 20. For the 40 min condition, the fracture surface exhibited a relatively rough and irregular morphology, with clear traces of adhesive deformation and stretching along the loading direction. These features indicate that the adhesive layer experienced noticeable deformation during loading, leading to fracture over a relatively wide area. In contrast, the 60 min condition showed comparatively smoother features with more localized fracture characteristics. The deformation traces were less pronounced, and the fracture appeared to occur within a relatively confined region. These observations are consistent with the higher load and energy absorption in the bonded region at 40 min shown in Figure 15 and Figure 17, reflecting the greater deformation of the adhesive layer compared to the 60 min condition.

**Figure 21:**
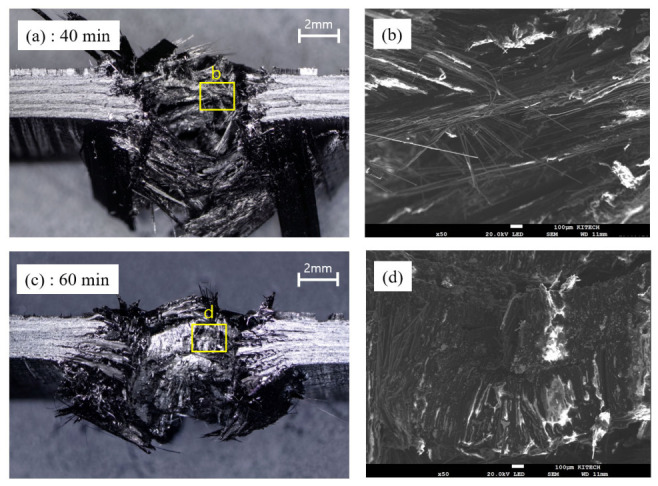
Fracture behavior in the riveted region at different curing conditions: (**a**,**b**) 40 min and (**c**,**d**) 60 min, with corresponding SEM images taken from the highlighted regions.

The fracture morphology in the riveted region exhibited distinct features, as shown in Figure 21. For the 40 min condition (Figure 21a,b), extensive fiber pull-out and pronounced deformation were observed around the rivet. The fibers were elongated and widely dispersed along the loading direction, indicating that sufficient deformation occurred in the riveted region prior to final failure. The SEM image in Figure 21b also shows long pulled-out fibers, suggesting that the applied load was gradually transferred through fiber pull-out and deformation. In contrast, the 60 min condition (Figure 21c,d) exhibited a more concentrated fracture morphology near the rivet, with a reduced degree of fiber dispersion compared to the 40 min condition. In particular, the lower region initially resisted the applied load, followed by a localized disengagement as the rivet was pulled out during failure. As shown in the SEM image in Figure 20b, the fiber pull-out length was relatively short and deformation traces were limited, indicating that the fracture occurred within a more confined region. These observations are consistent with the joint formation characteristics and mechanical behavior described earlier, where the reduced interlock at 60 min limited the load transfer capacity of the riveted region, resulting in localized deformation and premature pull-out failure, whereas the 40 min condition maintained sufficient interlock to sustain progressive deformation prior to final failure. These results indicate that the reduction in interlock with curing leads to a transition from progressive deformation to pull-out failure in the riveted region.

## 4. Conclusions

The influence of adhesive curing on joint formation and mechanical behavior of CFRP/aluminum hybrid joints was investigated, and the following are the main conclusions:

1. Five representative curing conditions of 0, 20, 40, 60, and 80 min were selected based on the curing-dependent lap shear response of the adhesive to represent the transition from the uncured state to the late-stage curing state. A sharp increase in the adhesive response was observed between 40 and 60 min, and a mechanical plateau was observed from 80 min.
2. Compared with the adhesive-only and riveting-only joints, the hybrid joint exhibited a two-stage load–displacement response, combining the high initial load of adhesive bonding with the sustained load over a larger displacement range provided by mechanical fastening.
3. The adhesive distribution changed with the curing time during the riveting process. As the curing time increased, the adhesive squeeze-out decreased, and the adhesive was increasingly retained near the contact point during riveting. At later curing times, the adhesive distribution changed from extensive squeeze-out in the uncured state to localized retention near the contact point, and the interfacial thickness near the center increased to approximately 0.28 mm at 80 min.
4. The adhesive’s curing-dependent resistance significantly altered the final joint geometry. As the curing time increased, the head height increased from 0.12 to 0.21 mm, whereas the interlock distance decreased from 0.67 to 0.54 mm. The increased adhesive resistance during riveting limits rivet penetration and suppresses radial expansion of the rivet legs, thereby changing the final joint formation.
5. In the bonded region, the peak load increased with curing time and reached 11.15 kN at 40 min, whereas in the riveted region, the load decreased from 4.13 to 3.42 kN at later curing times due to reduced mechanical interlocking. A maximum energy absorption of 32.13 J was observed at 40 min, where the joint exhibited relative contributions of the adhesive and the rivet. This condition was associated with bearing failure, indicating that curing-dependent changes in the adhesive behavior influenced the load transfer and the failure mode.
6. The present study focused on the effect of curing-dependent riveting behavior on mechanical performance under quasi-static loading. Therefore, future work will evaluate how curing conditions affect joint integrity under cyclic loading, thermal variation, and long-term environmental exposure in industrial applications.

## Figures and Tables

**Table 1:** Mechanical properties of sheets and rivets.

Material	Yield Strength (MPa)	Shear Strength (MPa)	Tensile Strength (MPa)	Elongation at the Break (%)	Hardness (Hv)
CFRP	-	120	825	1.8	-
A6061-T6	276	207	310	12	95
Rivet	960	690	1200	13	480

**Table 2:** Reaction heat and exothermic peak temperature of the adhesive at different curing times.

Curing Time (min)	Reaction Heat (J/g)	Exothermic Peak Temperature (°C)
0	242.89	102.61
20	127.47	109.04
40	96.96	116.64
60	49.49	127.71
80	32.34	135.00
100	29.63	139.50
120	21.56	143.50

**Table 3:** Reaction heat and exothermic peak temperature of the adhesive at different adhesive masses and curing times.

Curing Time (min)	Adhesive Mass (mg)	Reaction Heat (J/g)	Exothermic Peak Temperature (°C)
0	30	242.89	102.61
	20	263.94	101.05
	10	251.72	104.50
40	30	96.96	116.64
	20	87.66	123.50
	10	87.38	114.59
80	30	32.34	135.00
	20	28.85	140.51
	10	36.11	138.20

**Table 4:** Curing stage classification based on adhesive mechanical properties.

Curing Stage	Time (min)	Curing State	Shear Load (kN)
1	0	Uncured state	0
2	20	Early curing state	0.34
3	40	Intermediate curing state	0.55
4	60	Advanced curing state	8.50
5	80	Late-stage curing	9.26

## Data Availability

Data available on request due to restrictions (e.g., privacy, legal or ethical reasons).
